# Echoes of Past Contact: Venetian Influence on Cretan Greek Intonation

**DOI:** 10.1177/00238309221091939

**Published:** 2022-05-13

**Authors:** Mary Baltazani, John Coleman, Elisa Passoni, Joanna Przedlacka

**Affiliations:** Phonetics Laboratory, University of Oxford, UK; Department of Linguistics, Queen Mary University of London, UK; Department of Language and Linguistic Science, University of York; Phonetics Laboratory, University of Oxford, UK

**Keywords:** Language contact, intonation, Cretan Greek, Venetian, declarative and polar question tunes

## Abstract

Prosodic aspects of cross-linguistic contact are under-researched, especially past contact that has subsequently ceased. In this paper, we investigate declarative and polar question tunes of contemporary Cretan Greek, a regional variety of Greek whose speakers were in contact with Venetian speakers during the four and half centuries of Venetian rule on the island, from 1204 to 1669. The F0 contours of the Cretan tunes and alignment of peaks and troughs of interest with the nuclear vowel are compared to the corresponding tunes in Venetian dialect and Venetian Italian and to those in Athenian (Standard) Greek, which are used as control. The data (1610 declarative utterances and 698 polar questions) were drawn from natural speech corpora based on pragmatic criteria: broad focus for declaratives, broad focus, and information-seeking interpretation for polar questions. The pitch contour shapes of the tunes are modeled using polynomial basis functions, and the F0 alignment points are determined analytically. The results show the robustness of contact effects almost three and a half centuries after regular contact ceased and indicate that the shapes of the F0 contours of Cretan and Venetian declarative and polar question tunes are similar. In addition, Cretan alignment patterns are similar to Venetian and significantly different from Athenian. Insights are gained from research into how long prosodic characteristics may persist in a recipient language—decades or even centuries after the cessation of contact.

## 1 Introduction

Contact-induced linguistic influences are determined by the history of social relations among populations, including economic, political, and demographic factors. Long-term interactions between populations create multiethnic and multilingual communities, which in turn leads to the emergence of contact varieties (Sankoff, 2001). It is well known that cross-linguistic contact may impact all levels or aspects of language, with its effects on lexicon and morphology being well documented (see e.g., Clyne, 2003; Ralli, 2012; Thomason, 2001).

However, investigation of the prosodic aspects of language contact is a generally understudied area, although insights from such research are urgently required to deepen our understanding of resultant language change. McMahon (2004) draws attention to the status quo asserting that

the available evidence on the role of contact in phonological change is based virtually uniquely on segmental changes. It is possible that conclusions based on segmental developments will generalise straightforwardly to prosodic change, but it would be unwise to anticipate this without much more research. (p. 121)

This imbalance has been partly redressed through a number of more recent studies on prosodic variation in bilingual speakers (e.g., Elordieta & Calleja, 2005; Gabriel & Kireva, 2014; Lai & Gooden, 2018; Mennen, 2004; O’Rourke, 2012; Queen, 2012; Romera & Elordieta, 2013; Simonet, 2010; Van Rijswijk & Muntendam, 2014). Broadly, the findings demonstrate that the intonation of bilingual speakers may contain characteristics of both their languages. For example, Queen (2012) found that terminal rises in bilingual Turkish-German speakers living in Germany are realized with two distinct patterns, one similar to German and the other to Turkish. Along the same lines, O’Rourke (2012) showed that there is later peak alignment in the intonation of contrastive focus in the speech of Cuzco (Peruvian) Spanish speakers who were in close contact with Quechua, in comparison to Lima Spanish speakers who had less contact with Quechua. The peak alignment pattern of Cuzco Spanish speakers resembled the Quechua’s later alignment pattern, in contrast to the early peak alignment pattern of Lima Spanish speakers.

Similar results have been reported for Basque speakers of Spanish (Elordieta, 2003; Elordieta & Calleja, 2005; Elordieta & Irurtzun, 2016), in that L1 Basque speakers in urban areas such as Bilbao show late prenuclear peak alignment, similar to Madrid Spanish, while Lekeitio L1 Basque speakers show early peak alignment as a result of the transfer of the prenuclear Basque pattern. Colantoni and Gurlekian (2004) describe the intonation patterns of early peak alignment in Buenos Aires Spanish as a result of convergence between the Spanish and the Italian intonation systems. Mennen (2004) reports that the pattern of peak alignment in the speech of Dutch speakers of Greek as L2 is intermediate between the two languages.

In addition, recent studies have shown that contact between typologically distinct languages may give rise to different varieties, each with its own prosodic system, as for example, in post-colonial varieties of English and French (Gut, 2005; Payne & Maxwell, 2018; Zerbian, 2013). All in all, the literature gives support to the idea that ongoing language contact as experienced by bilingual and L2 speakers results in intonational variation and change, giving rise to novel patterns that may combine elements from both contextual languages.

On the other hand, the effects of language contact on the diachronic development of intonation are less well studied, although evidence is emerging that prosodic characteristics may persist in a recipient language for decades or even centuries after the cessation of contact. This has been reported for the intonation of Buenos Aires Spanish, influenced by contact with Italian in the 1850s (Colantoni & Gurlekian, 2004); in present-day Asia Minor Greek after centuries-long contact with Turkish, which ended in 1923 (Baltazani et al., 2019a, 2019b); in Pennsylvania English spoken in Frenchville, after the end of contact with French in the 1830s (Bullock, 2009); and in New Mexico English, with its Hispanic intonation patterns relatively stable over the 20th century, despite the change from a Hispanic majority to a Hispanic minority in New Mexico during this period (Van Buren, 2017). Often the contact-induced prosodic patterns co-occur with corresponding patterns from the dominant language (Bullock, 2009) but with distinct pragmatic uses. The theoretical interest in the diachrony of intonation is self-evident not only on its own merit but also as a means of understanding prosodic cross-dialectal variation and the typology of intonation more generally.

The work reported here is part of a project^1^ that aims to understand how long-term cross-linguistic contact has contributed to regional differences in the intonation of contemporary varieties of Modern Greek, which were formerly in contact with Turkish (Asia Minor Greek and Cypriot Greek), Venetian (Corfu Greek), or both (Cretan Greek). In the present paper, we compare declarative and polar question intonation tunes in Cretan Greek with their counterparts in Venetian dialect and Venetian Italian (henceforth “Venetian” as their intonation contours are not significantly different we subsume these varieties under the single label; see section 3) to discover possible influences of the latter on the former. Standard Modern Greek as spoken in Athens (henceforth Athenian) is used as a control. We present evidence that the declarative and yes-no question tunes in Cretan Greek are not significantly different from those in Venetian and significantly different from their Athenian counterparts. We quantify those similarities and differences by a combination of tools: the first is mathematically modeling the shape of the tunes employing techniques of Functional Data Analysis (Ramsay et al., 2010; see 2.2 for details of the methods) and the second is using mainstream Autosegmental-Metrical (Ladd, 2008; Pierrehumbert, 1980) measures such as the alignment of specific F0 turning points in the tunes with relevant segmental landmarks. Unlike most current intonation studies, we take a corpus-based approach (Taylor, 2008), using statistical methods to discern patterns in corpora of natural speech in the three varieties.

### 1.1 Historical background on Cretan—Venetian contact

During the era of the Republic of Venice, several areas of Greece were under Venetian dominion at different times. The island of Crete, which bounds the southern border of the Aegean Sea in the Eastern Mediterranean, remained under Venetian rule for four and a half centuries, from 1204 to 1669. Throughout this period, many sociolinguistic circumstances were in place that might have supported prosodic transfer (Holton, 1991; Watrous, 1982). For example, the Venetian settler policy meant that as early as the end of the 13th century approximately one-sixth of the 10,000 inhabitants of Crete were Venetian settlers (Watrous, 1982). This resulted in close Greek–Venetian language contact, essential for the trade of agricultural products (Stallsmith, 2007, p. 153) and administration (Maltezou, 1991), where, as a common practice of the Venetian government, Greek language documents were written in the Latin script (Manolessou, 2018, p. 156). Cretan letters and arts of the time such as painting, architecture, and music flourished, resulting in exchanges between the two cultures (Holton, 1991). Moreover,

Early in the Venetian period, intermarriage between Cretan archontic families and Venetian nobles, as well as irregular unions between Venetians and lower-status Cretans, fostered a Veneto-Cretan culture. That Venetian colonial nobles joined native Cretans in the 1363 revolt of St. Titus indicates how early this cultural synthesis had begun. (Stallsmith, 2007, p. 156)

In 1669, the Ottomans conquered Crete and the island remained under their occupation until 1898 when it became an autonomous state. The political and administrative organization of Ottoman rule differed vastly from the Venetian one and so did the resultant social makeup of the Cretan population at the time. Unlike Venetians, Ottomans did not transfer colonists to Crete (Greene, 2000, p. 87; Hooper, 2003, p. 27; Stallsmith, 2007, p. 161), resulting in a less systematic and more decentralized administration. Although a large proportion of the indigenous monolingual Cretans converted to Islam, many did so for the sake of socioeconomic advantages (Hayden, 2004, p. 299): as Muslims, they were allowed to purchase and hold on to their land, which under the Ottoman ideology would otherwise have been taken and made available to Ottoman officials and supporters (Stallsmith, 2007, p. 161). Apart from religious conversion, however, little mixing of populations or cultures ensued.

Throughout the Ottoman occupation, Crete maintained contacts with Venice through trade (Greene, 2000, p. 128) and imports such as textiles and glass from Venice (Greene, 2000, pp. 126–127). It is notable that in the *Linguistic Atlas of Crete* (Kontosopoulos, 1988), for example, there are far more lexical items borrowed from Venetian than from Turkish, evidence that Venetian-influenced Cretan Greek was maintained throughout the Ottoman period. Recent recordings of Cretans are found in the *Greek Dialects Archive*, a database of around 2500 linguistic field recordings from the School of English, Aristotle University of Thessaloniki recorded between 2006 and 2018 (http://www.enl.auth.gr/greekdialects/preface.htm) is also a testament to the survival of Venetian intonational melodies through oral culture, that can be heard in recitations of traditional folk song lyrics and poetry, such as *Erotokritos*, a 17th-century Cretan romance.

After the end of the Ottoman occupation, Crete remained an autonomous state, separate from mainland Greece. While Greece was recognized as a sovereign state in 1830, Crete joined the Hellenic Republic much later. The Greek government declared Crete a Greek territory in 1912, but this was internationally recognized in 1913.

### 1.2 Linguistic background: Athenian, Venetian, and Cretan

Athenian, the Modern Greek variety spoken in Athens, is the standard used for public purposes, in education, and in the media. Athenian is thought not to be distinctly marked by any single traditional Greek dialect (Kontosopoulos, 1999; Trudgill, 2003). Extensive internal migration from various rural parts of the country to Athens between 1950 and 1980 (Allen, 1986) resulted in today’s Athenian, an amalgamation of different varieties brought by migrants who moved to the capital and its surroundings.

Cretan Greek, spoken mainly on the island of Crete, is typically divided into two geographical subvarieties, Eastern and Western (Kontosopoulos, 1988; Ksanthinakis, 2001; Lengeris et al., 2014), mainly based on morphological and segmental phonetic differences. No intonational differences between the two varieties have been reported. Τhere is evidence that the Cretan dialect diverged from the language spoken in areas under Byzantine rule (i.e., mainland Greece) around the late-14th century and that the modern form of this dialect solidified in its written form in the late-16th century (Horrocks, 2010, pp. 360–361). According to Horrocks, several important phonological and morphological changes in Cretan coincided with the social changes during this time of Cretan-Venetian cohabitation.

The third variety examined in this article is Venetian, spoken in the lagoon and mainland areas of the city of Venice (Zamboni, 1974). Venetian has the Venetan dialect as a substrate, which was a Western Romance variety descending from vulgar Latin, and the spoken (and for a time written) language of the Republic of Venice (Ferguson, 2003, 2005). The linguistic reality of the opposition of modern regional Italian to dialect is complex (Cerruti et al., 2017). There is no clear demarcating line between the two but rather a continuum, due to a situation of continuous language contact. Our corpus contains utterances along this continuum, which we initially classified into three categories: Venetian Italian and Venetian Dialect as the two ends of the continuum, and Italianized Venetian in between the two. This classification was based on a set of lexical, morphological, and segmental criteria set out by Canepari (1976) and Ferguson (2007); see Appendix B for a list of the criteria. The term Italianized Venetian is used in work on the convergence between the dialects and Italian (Grassi, 1993).

### 1.3 Intonational background: Athenian, Venetian, and Cretan

Very little is known about intonational variation in Greek (but for variation in Athenian Greek, see Baltazani et al., 2020; Gryllia et al., 2018; Lohfink et al., 2019). Even less is known about the intonational systems of regional varieties of Greek, bar a handful of studies involving a single tune or phenomenon (e.g., Arvaniti, 1998, on Cypriot Greek polar questions; Baltazani & Kainada, 2015, 2019, for Epirus and Cretan Greek declaratives, respectively; Giakoumelou & Papazachariou, 2013, on Corfu polar questions; Papazachariou, 1998, 2004, on Northern Greek polar questions; Papazachariou & Archakis, 2001 for polar questions in the dialect of Goumenitsa in Northern Greece; Themistokleous, 2012 for Cypriot Greek declaratives).

In comparison, more work has been done on several Italian regional varieties (Avesani, 1990; Caputo & D’Imperio, 1995; D’Imperio, 2002; Gili Fivela et al., 2015; Grice, 1995). Nonetheless, research on the intonation of northern Italian varieties in general and the varieties spoken in the Veneto region in particular is scarce (but see Savino, 2012 for analysis of Venetian polar questions; Payne, 2005 for Trevigiano dialect; Canepari, 1986 and Dévis-Herraiz, 2007 for Venetian declaratives and polar questions).

In the remainder of this section, we present the descriptions of declarative and polar question tunes in each of the three language varieties according to the literature, illustrated by examples drawn from our own corpora. For tunes that have not been investigated hitherto, we offer an initial, impressionistic description, that will be refined in more quantitative terms in later sections.

**Figure 1.**
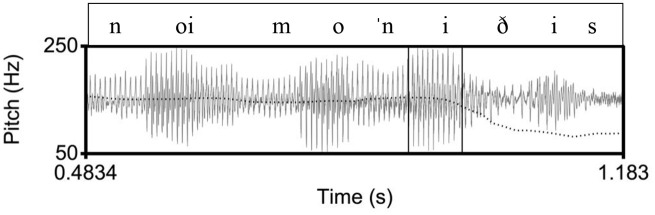
Example of a declarative utterance in Athenian, ([le ɣˈotan] noimoˈniðis) “(He was called) Noimonidis.” The inner rectangle indicates the stressed vowel on [ni] in the nuclear word [noimoˈniðis]. In the Athenian tune, there is a high pitch during the stressed vowel and a low pitch after the stressed vowel.

Broad focus declarative utterances were chosen for all three language varieties, which indicate that an accented word is new in discourse. In Athenian, the tune of these utterances is characterized by a peak aligned with the nuclear vowel, followed by a fall. This broad focus pragmatic function can be expressed through two tunes in Athenian, one which may be described in ToBI notation as H* L-L% (Arvaniti & Baltazani, 2005; Baltazani, 2002, 2003; Lohfink et al., 2019; Figure 1) and the other as H*+L L-L% (Arvaniti & Baltazani, 2005; Lohfink et al., 2019). The difference between the two, claimed to be one of pragmatic interpretation, has not been conclusively determined. While some studies claim that the interpretation of H*+ L (in addition to marking the accented word as new in discourse) also conveys a sense of “stating the obvious,” that is, the implication that the addressee should have known or expected the answer (Arvaniti & Baltazani, 2005; Lohfink et al., 2019), this claim has not been confirmed by other studies (Baltazani & Kainada, 2019), where the pragmatic interpretations of the H* and the H*+ L accents have been reported to overlap. In the present article, we do not discriminate between those two tunes and examine all broad focus declaratives in our corpus. These two tunes have been experimentally shown to be shared by other Greek dialects, for example, Cypriot Greek (Themistokleous, 2012) and Epirus Greek (Baltazani & Kainada, 2015).

**Figure 2.**
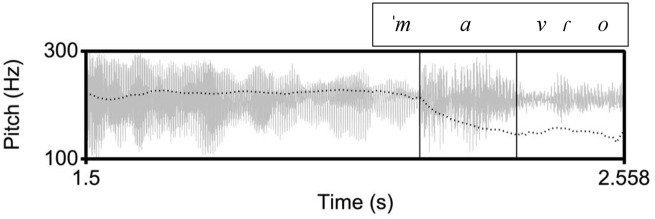
An example of a declarative utterance in Cretan, ([to ˈena ˈine ɣalaˈno tʃe] to ˈalo ˈine ˈmavɾo) “(One is blue and) the other is black.” The inner rectangle indicates the stressed vowel on [ma] in the nuclear word [ˈmavɾo]. In the Cretan tune, there is a high pitch before the stressed vowel and a low pitch on the stressed vowel.

**Figure 3.**
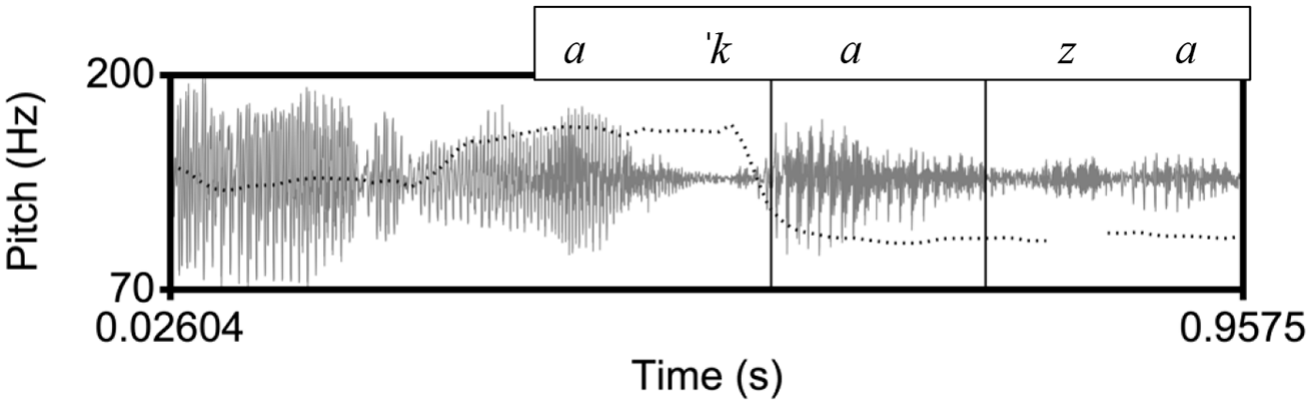
An example of a declarative utterance in Venetian, [ˈtorno a ˈkaza] “I go back home.” The inner rectangle indicates the stressed vowel on [ka] in the nuclear word [ˈkaza]. Here, as in the Cretan example, there is a high pitch before the stressed vowel and a low pitch on the stressed vowel.

The Cretan Greek broad focus declarative tune is also a fall (Figure 2); however, impressionistically it sounds markedly different from the Athenian one. In a preliminary study based on a small corpus of 27 tokens, this tune was described as H + L* L-L%, in which a trough is aligned with the nuclear vowel, followed by a low plateau (Baltazani & Kainada, 2019). More remarkably, the Cretan fall sounds impressionistically similar to the Venetian declarative fall (Figure 3), described as L* L-L% in Venetian by Dévis-Herraiz (2007), which also ends in a fall.

**Figure 4.**
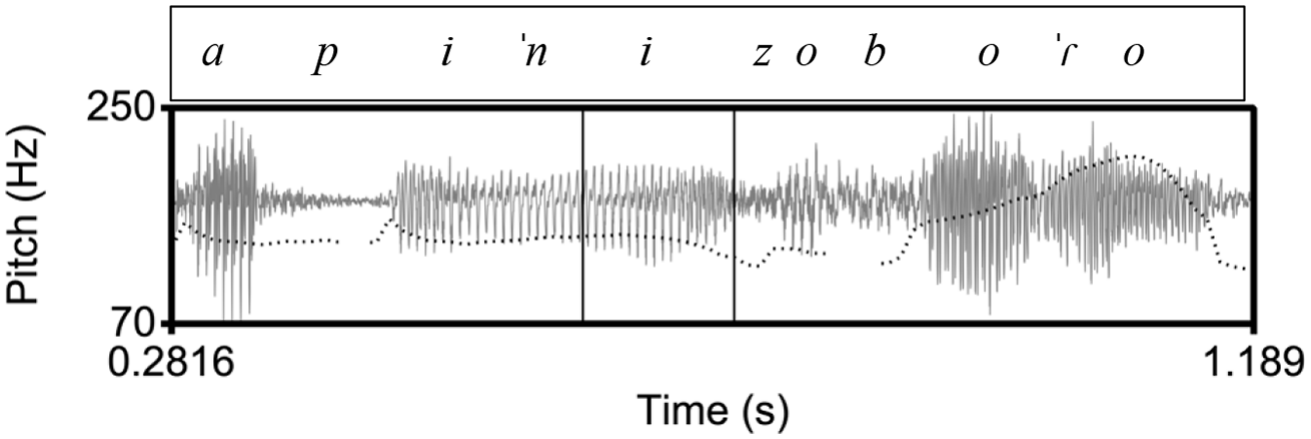
An example of a polar question in Athenian, ([ce na] kapiˈnizo boˈɾo) “Can I (also) smoke?” The inner rectangle indicates the stressed vowel on [ni] in the nuclear word [kapiˈnizo]. In the Athenian tune, there is a low pitch during the stressed vowel followed by a rise-fall.

Polar questions in Athenian, Cretan, and Venetian can be phonetically identical to statements, that is, there is no morphological marking or word order differences so that statements are only distinguished from polar questions through intonation. The tune of polar questions in Athenian has been described as L* LH- L% (Arvaniti et al., 2006; Baltazani, 2007). In it, the nucleus aligns with a trough (Figure 4) followed by a rise-fall.

**Figure 5.**
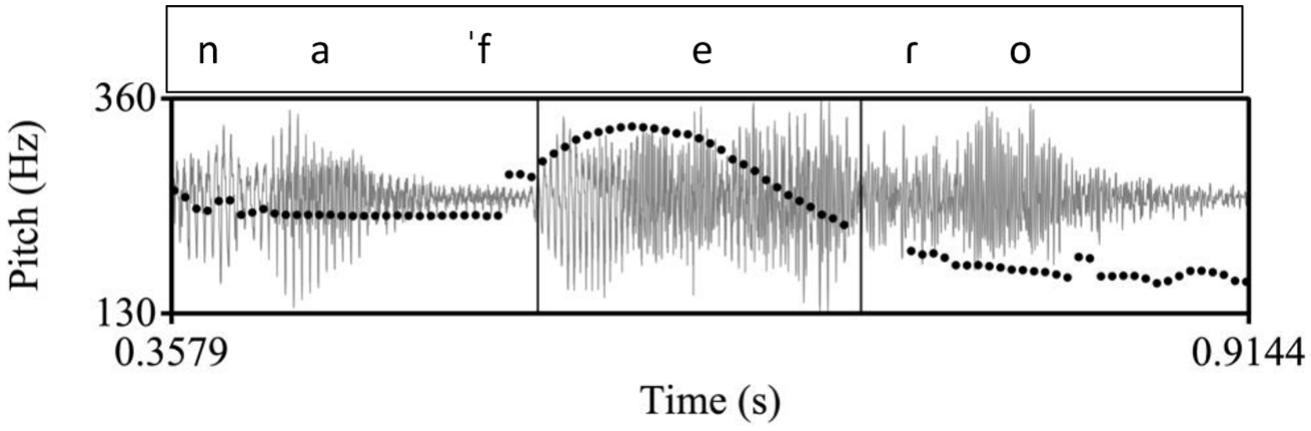
An example of a polar question in Cretan, ([kaˈɾekla] na ˈfeɾo) “(chair) shall I bring?.” The inner rectangle indicates the stressed vowel on [fe] in the nuclear word [ˈfeɾo]. In the Cretan tune, there is a high pitch during the stressed vowel followed by a fall.

The Cretan polar tune (Figure 5) has not been investigated so far to our knowledge. Impressionistically, it is quite different from the Athenian tune. During the accented vowel there is a peak instead of the Athenian low F0 and the question ends in a fall instead of the Athenian rise-fall.

**Figure 6.**
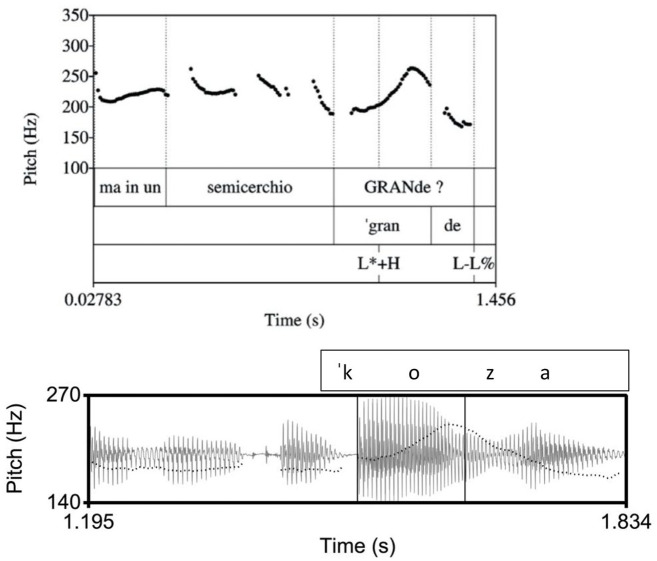
Top: Venetian polar question from Savino (2012, p. 39), showing the nuclear pitch as a peak over the stressed syllable in the word [ˈgrande]. Bottom: Example of a polar question in Venetian from our corpus, [ai kwalˈkoza] “is there anything?.” The inner rectangle indicates the stressed vowel on [ko] in the nuclear word [kwalˈkoza]. In the Venetian tune, in both panels, there is a high pitch during the stressed vowel followed by a fall.

In Venetian, the polar question tune has been described as an accentual rise followed by a fall (Figure 6 top; Savino, 2012). Impressionistically, the data from our corpus confirm this description (Figure 6 bottom).

## 2 Method

### 2.1 Materials and speakers

Unlike most Autosegmental-Metrical studies of intonation that analyze laboratory speech and controlled materials, we follow the practice of many language contact studies and draw on natural speech corpora obtained from a number of sources (see Appendix A) for our analysis. This is because many of the sociolinguistic parameters that drive the behavior of speakers are not well understood and cannot be replicated in the laboratory. The recordings in our corpora vary in length from a few minutes to an hour and come from various preexisting sources of spontaneous (e.g., television and radio interviews, linguistic fieldwork interviews, dialogues, and narratives) and semi-spontaneous speech (map task, “spot the difference” task, and recitations) created between 2001 and 2019. This material comprises a variety of speech styles, including short reading passages, sentence lists, and sociolinguistic interviews with one or more informants. Single utterances were extracted from them (see section 2.2 for the selection criteria), which also vary in length and syntactic structure.

Table 1 presents the number of declarative and polar question tune tokens analyzed per language variety, showing the number of speakers as well as their sex and age distribution. Overall, the sample analyzed in this study comprises 1,610 tokens of the declarative tune and 698 tokens of the yes–no (polar) question tune produced by 73 and 60 speakers, respectively.

**Table 1.** Number of Tokens per Tune and Language Variety, Together with Sex and Age Distributions.

Language Variety	Declarative tokens	Male speakers	Age range	Female speakers	Age range
Athenian Greek	324	12	27–69	9	25–60
Cretan Greek	447	19	37–93	12	35–83
Venetian	833	10	18–65	11	18–40
Language Variety	Polar question tokens	Male speakers	Age range	Female speakers	Age range
Athenian Greek	273	6	40–86	6	35–82
Cretan Greek	135	17	32–84	14	35–83
Venetian	288	9	18–30	8	18–30

### 2.2. Procedure

The first step in our investigation, as demonstrated in 1.2, involved an impressionistic phonological analysis based on a small subset of the data for each language variety. Next, the insights gained from the first step were used as a basis for mathematical modeling. We employ techniques of Functional Data Analysis (Ramsay et al., 2010), a statistical approach to analyzing continuous data such as curves, signals, or surfaces used in a broad spectrum of disciplines such as physiology (growth curves), demographics (population variables), weather forecasting, and more recently speech (Andruski & Costello, 2004; Grabe et al., 2007; Zellers et al., 2010).

Functional data analysis begins with data smoothing, a process that converts raw discrete data points (in this case measured F0 values) into a smoothly varying function. This emphasizes patterns in the data by minimizing short-term deviation due to measurement errors or inherent system noise. Functional data also allow using the information on the derivatives (rates of change) of the curves, which facilitates comparisons across languages, language varieties, genders, generations of speakers, and other sociolinguistic or phonetic variables. A significant advantage of functional data analysis is that it augments the highly abstract and impressionistic Autosegmental-Metrical analysis of intonation with quantitative, empirically testable models of tunes, allowing comparisons of large numbers of pitch curves of whole utterances or their parts in corpora and facilitating the study of melodic variability, co-occurrence patterns of tone combinations in melodies, and frequency of occurrence.

All our data sources were digital, but they came in a wide variety of formats (e.g., MP3, MP4, and WAV; 2-channel or mono), bit rates, or sampling rates (e.g., 44.1, 22.05, or 16 kHz). In addition, some digital recordings made from ¼-inch tape recorded at different speeds required speeding up or slowing down. A small number of such digitized tape recordings ran backward on one channel as if the tape spool had originally been turned over for the second half of the recording, but it had been digitized as if it were a 2-track stereo recording. To permit for the subsequent functional data analysis steps to be performed as batch computations, we converted all the recordings to 16 kHz, monophonic, uncompressed PCM .wav audio files.^2^

We employed pragmatic criteria for choosing the utterances we would analyze in all three language varieties to avoid the circularity that would ensue had we based our selection on intonational features. For declaratives, as already mentioned, we chose broad focus utterances in which no constituent carries a narrow focus. Similarly, for the polar tune, we selected questions with a broad focus intonation structure and an information-seeking pragmatic interpretation (Caponigro & Sprouse, 2007). Information-seeking questions can be distinguished from confirmation-seeking and rhetorical questions in their pragmatic interpretation based on context and have been shown to differ in their intonation in several languages (e.g., Grice et al., 1995; Vanrell et al., 2010).

The sound files were orthographically transcribed, translated into English, and segmented into utterances. For each variety, a native speaker identified the relevant utterances, located the nuclear word, and manually annotated the beginning and the end of the stressed vowel in Praat (Boersma & Weenink, 2018). The beginning of the nuclear vowel was used as a phonological landmark to delimit the start of a region of interest on which our modeling was based (see section 2.3). The end of the stressed vowel was used as the segmental landmark used to calculate the differences in the alignment of H peaks or L troughs in the three language varieties.

**Figure 7.**
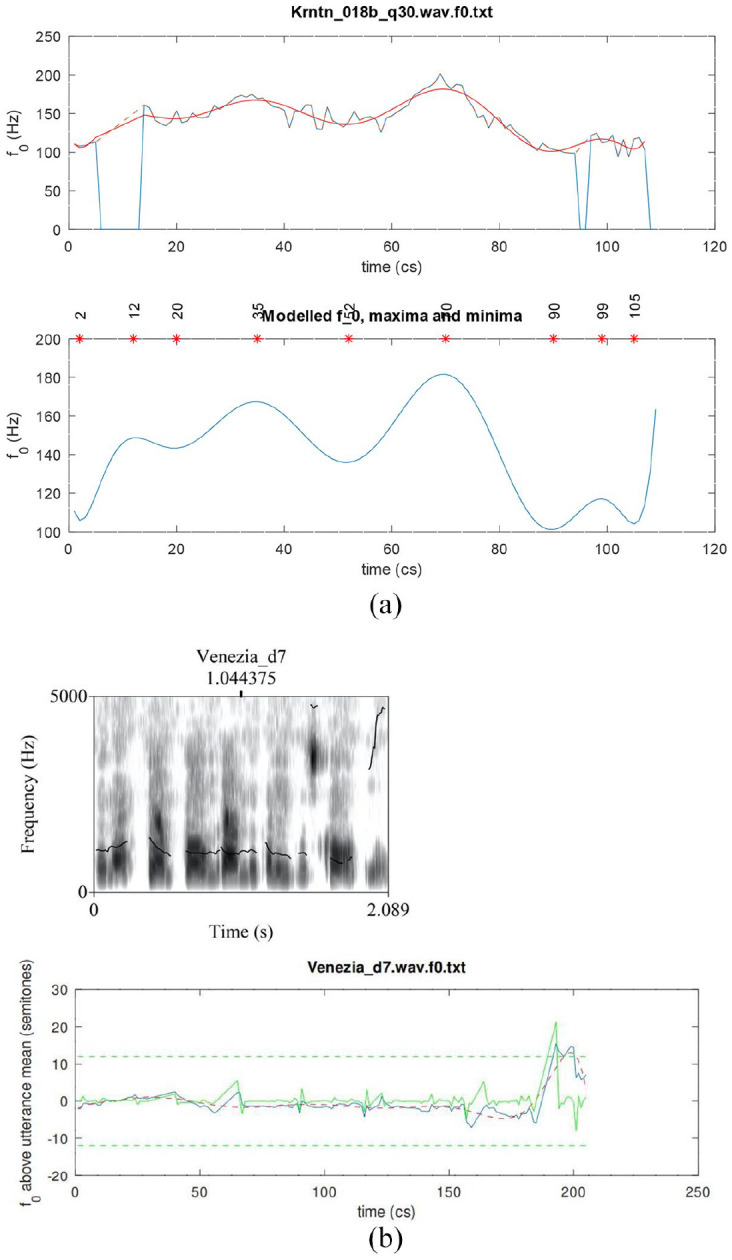
(a) An example of a 10th-order polynomial fitted to the F0 contour of an utterance in our corpus. Top: the smooth modeled curve is superimposed over the observed curve that is characterized by rapid pitch perturbations due to consonantal onsets or offsets. Note also the two voiceless stretches in the observed curve, indicated by dashed lines at the beginning and near the end of the utterance. Bottom: The modeled F0 maxima and minima locations, indicated by the stars. (b) An example of an octave error (pitch doubling). Top: Praat spectrogram with a pitch track. Bottom: The observed curve (continuous line), the modeled curve (dashed line), and the errors curve (green line). Errors are indicated by a spike reaching the green horizontal dashed line at the top and/or bottom of the panel (showing a difference of ±12 semitones). For each utterance, we verified whether this is a pitch estimation error or a genuine doubling or halving of the pitch in the signal, taking into account acoustic visual and auditory information, as well as the F0 in the entire utterance.

For each utterance, F0 was measured every 10 milliseconds using the ESPS (Entropics Signal Processing System) *get_f*0 function (Talkin, 1995), to obtain an F0 time series. The 10th-order polynomials

(1) $$F0_{\mathrm{fitted}}=\;\Sigma a_{n}t^{n}\mathrm{for}\;n\;=\;0,\;\ldots ,\;10,$$

were fitted to F0 contours using GNU Octave *polyfit* function (Figure 7(a) shows an example of a 10^th^-order polynomial fitted to the F0 contour of an utterance in our corpus). A 10th-order polynomial was used to fit sentences that may be very long, with very many (up to 9) peaks and troughs.

To identify F0 tracking errors (especially octave doubling and halving), and to provide a degree of normalization of between-speaker differences in voice pitch, the F0 contours of each utterance were converted to semitones above or below the whole utterance mean F0, as in Equation 2: This makes every token directly comparable to all the others and goes some way to normalizing for interspeaker differences. If the data had included better information about speaker identities, we might preferably have transformed F0 to semitones above or below the *speaker* mean, but we lack that data.

(2) $$F0\left[\mathrm{semitones}\right]\;=\;12/\log_{10}\left(2\right){\;\log}_{10}(F0\left[\mathrm{Hz}\right]/F0\left[\mathrm{Hz}\right]).$$

Given this transformation, an octave error is a sudden jump of exactly 12 semitones (Figure 7(b)). All such potential octave jumps were individually inspected to check that they were octave errors not actually occurring pitch changes; those error portions were then corrected by doubling or halving the F0 measurements, as appropriate. Other large or sudden jumps in F0 were similarly inspected to determine their cause. Rapid pitch perturbations due to consonantal onsets or offsets were retained unaltered. In other cases, rapid jumps could be attributed to environmental noises or to other speakers; such portions were excluded from the measured F0 tracks.

Fitting a specific function to the data also permits us to find the peaks and troughs in the F0 contour analytically, by differentiating Equation 1 as they lie at points where its first derivative (3) is zero:

(3) $$\mathrm{dF}0/dt=\;10a_{10}t^{9}+{\;9a}_{9}t^{8}+\;\ldots \;+{\;a}_{1}.$$

The roots (values of *t*) at which dF0/d*t* = 0 were found using the Octave/MATLAB function *real(roots(polyder(a))).* These roots give us the times of all the maxima and minima (peaks and troughs) of the intonation contour (Figure 7(a)). Putting any of these times back into Equation 1 gives the F0 of that peak or trough.

Since there are 9 such turning points in a 10th-order polynomial, we then manually selected the local minimum after the start of the nuclear vowel in declaratives and the local maximum after the start of the nuclear vowel in polar questions. That is, we located the peak after the beginning of the vowel for polar questions and the trough after the beginning of the vowel for declaratives. We used those maxima and minima to determine the alignment of turning points to segmental landmarks.

Thus, polynomial modeling enables us to calculate very precisely the location of peaks and troughs in the F0 track. We note that prior work on intonation has often found it difficult to estimate the location of such turning points due to the presence of voiceless stretches or microprosodic perturbations (see, for example, the voiceless stretches in Figure 7(a)), finding recourse instead in specially designed laboratory utterances containing mostly sonorant segments. We also note that, as explained in Kochanski (2010), simply picking maxima and minima of observed, measured F0 is a rather questionable and inexact method. The location of a high or low F0 “target” might happen to coincide with a portion of voicelessness in the speech signal, in which case the observed F0 contour will not even have a value at such a point. In contrast, the method used here is well able to infer a highly accurate estimate of the peak or trough location and estimate an F0 value even during intervals of voicelessness (see, for example, the maximum at 12 cs from the start of the utterance in Figure 7).

### 2.3 Data modeling

A Region of Interest was defined as the interval from the beginning of the stressed vowel in the nuclear word to the utterance end. This interval contains from one to three syllables, depending on the variety and stress position (antepenultimate—penultimate—final).

**Figure 8.**
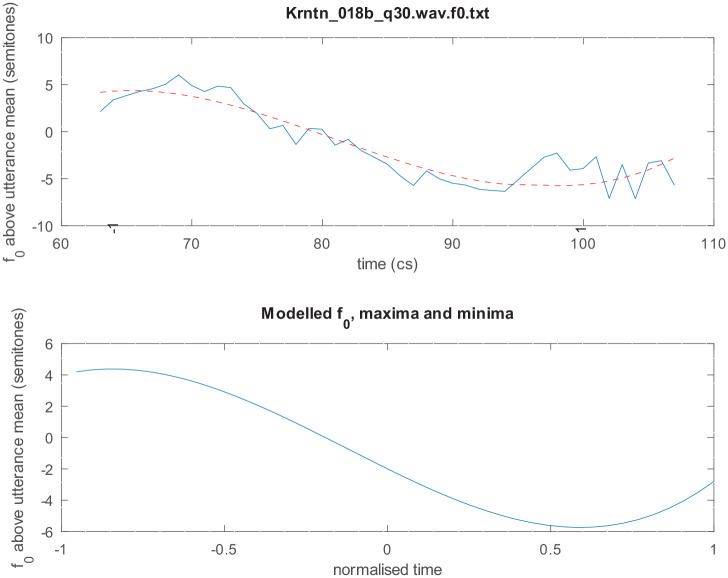
The cubic fitting of the region of interest of the same utterance is depicted in Figure 7(a).

To characterize similarities or differences in the overall shapes of the selected tunes, we modeled the sampled F0 data using polynomial basis functions, following Grabe et al. (2007). This process converts discrete data points (measured F0 values) into a smoothly varying, continuous function, for example,

(4) $$F0={\;a}_{1}t^{3}+{\;a}_{2}t^{2}+{\;a}_{3}t+{\;a}_{4}+\varepsilon ,$$

for the cubic case (Figure 8), which is but one among various possibilities. The values of constants a_1_ to a_4_ were found using the Octave/MATLAB *polyfit* function.

The fitted polynomial (4) was then converted into the corresponding form (5), the best-fitting sum of Legendre polynomials each of which is normalized to have unit variance.

(5) $$F0_{\mathrm{fitted}}={\;c}_{1}L_{3}+{\;c}_{2}L_{2}+{\;c}_{3}L_{1}+{\;c}_{4}L_{0}$$

This transformation has the benefit that the c_n_ coefficients of each term are orthogonal, unlike the a_n_ coefficients of the conventional polynomial (4). Low-ranking polynomials pick out slowly varying properties and the higher-ranking polynomials pick out successively more rapidly varying properties. Legendre polynomial L_0_ models the average of a delimited extract of data, L_1_ models the slope of the extract, L_2_ fits a parabola to the data, and L_3_ models the data as an up-down-up (or down-up-down) wavy shape. If the sign of the respective c_n_ coefficient is inverted, those patterns are flipped upside-down about the horizontal axis; see Grabe et al. (2007) for further details.

### 2.4 Hypotheses

Our general hypothesis, driven by findings in the literature as well as the impressionistic analyses given in 1.3, is that Cretan declarative and polar question tunes will display similar intonational characteristics to Venetian Italian, both in the shape of the F0 curves, as revealed by the fitted polynomial coefficients (see 2.3), and the alignment of the relevant turning points. We chose to compare the alignment of turning points that all three varieties had in common for each tune, that is, the H and L tones in declaratives and the H peak in polar questions (see section 4).

Specifically, we expect that in the declarative tunes there will not be differences in the shape characteristics of the F0 curves because in all three language varieties the declaratives end in a fall. Instead, we expect to find differences in the location of the F0 peak (corresponding to an H tone) and the F0 trough (L tone). The H is expected to align with the nuclear vowel in Athenian (as in Figure 1) because it is the nuclear accent, but it is expected to occur before the nuclear vowel in Cretan (Figure 2) and Venetian (Figure 3); for both Cretan and Venetian, we hypothesize this H tone is a leading tone of a bitonal H + L* pitch accent. On the other hand, the L is expected to align with the nuclear vowel in Cretan and Venetian because it is the nuclear accent but to occur after the end of the nuclear vowel in Athenian because it is part of the edge tones (see section 1.3).

More differences are expected in the polar question tunes, where both the shape characteristics of the F0 curves (especially the cubic and quadratic coefficients) and the alignment of the peak are expected to be similar in Cretan and Venetian and different in Athenian from the other two (Figures 4 to 6). In particular, we expect the Cretan and Venetian shape to be a rise-fall (convex upward), while the Athenian shape is expected to consist of a trough followed by a rise-fall (a wave-like shape). We also expect the F0 peak to align with the nuclear vowel in Cretan and Venetian because it is the nuclear accent but to be considerably later than the end of the nuclear vowel in Athenian because it is part of the edge tones (see 1.3, Figures 4 and 5).

A further hypothesis relates to the continuum of varieties spoken in the Venice area (see the discussion in 1.2): despite the morphological, lexical, and segmental differences between the varieties spoken in the Venice area, we expect no significant difference in the intonation patterns between the three subgroups defined in 1.2. This hypothesis is based on claims in the literature that the intonation of the substratum dialect often percolates into the Regional Italian variety, for example:

. . . [la] prosodia e [l’] intonazione, [sono i] più potent[i] marcator[i] di caratteristiche regionali. . . Telmon (1993, p. 103)

(Our translation: Prosody and intonation are the strongest markers of regional speech traits. . .)

. . . spesso coloro che hanno eliminato le caratteristiche articolatorie più marcatamente regionali della loro pronuncia, conservano le strutture intonative della loro parlata originaria: ché sono le più difficili da modificare . . . Canepari (1979, p. 226)

(Our translation: Often those who succeeded in removing from their speech articulatory features typical of their region of origin still maintain the intonation of their original variety, which is the more difficult feature to modify . . .)

To test this hypothesis, we compare the Venetian Dialect and Italianized Venetian tokens with Venetian Italian tokens in the third section.

To examine differences in the *shape* characteristics of the F0 contours (in 4.1.1), as well as differences in the *alignment* of L troughs in declaratives (in 4.1.2) and H peaks in polar questions (in 4.2) with segmental landmarks, we carried out Kruskal–Wallis one-way analyses of variance with each of the first four coefficients as well as the alignment values as dependent variables and *language variety* (with three levels: Athenian, Cretan, Venetian) as the independent variable^3^ (Kruskal & Wallis, 1952). A similar Kruskal–Wallis one-way analysis of variance was used for the comparison among the three Venetian varieties in the third section. A non-parametric test was used for all the analyses mentioned earlier because it is suitable for unequal sample sizes and unequal variances. Dunn’s post hoc pairwise tests were carried out for all pairs of language groups. For the more detailed analysis of the H and L tone alignment in declaratives^4^ (see 4.1.2), univariate analysis of variance (ANOVA) tests (suitable for equal sample sizes) were used, with the H and L alignment times as dependent variables and language variety as the independent variable (with five levels: Athenian, Cretan, Venetian Italian, Italianized Venetian, and Venetian Dialect); Tukey honestly significant difference (HSD) pairwise tests were carried out for all the pairs of language groups to identify any significant differences.

## 3 Comparison among the Venetian varieties

### 3.1 Motivation

As explained in Sections 1.2 and 2.4, our corpus contains tokens from three varieties spoken in the Venetian area, labeled Venetian Dialect, Italianized Venetian, and Venetian Italian. In this section, we test the shape characteristics and alignment of the L tone in declaratives between these three Venetian varieties, to detect possible differences. If no statistically significant differences are detected, the three Venetian varieties will be treated as one group that will be compared to Cretan and Athenian in 4.

### 3.2 Comparison results

**Figure 9.**
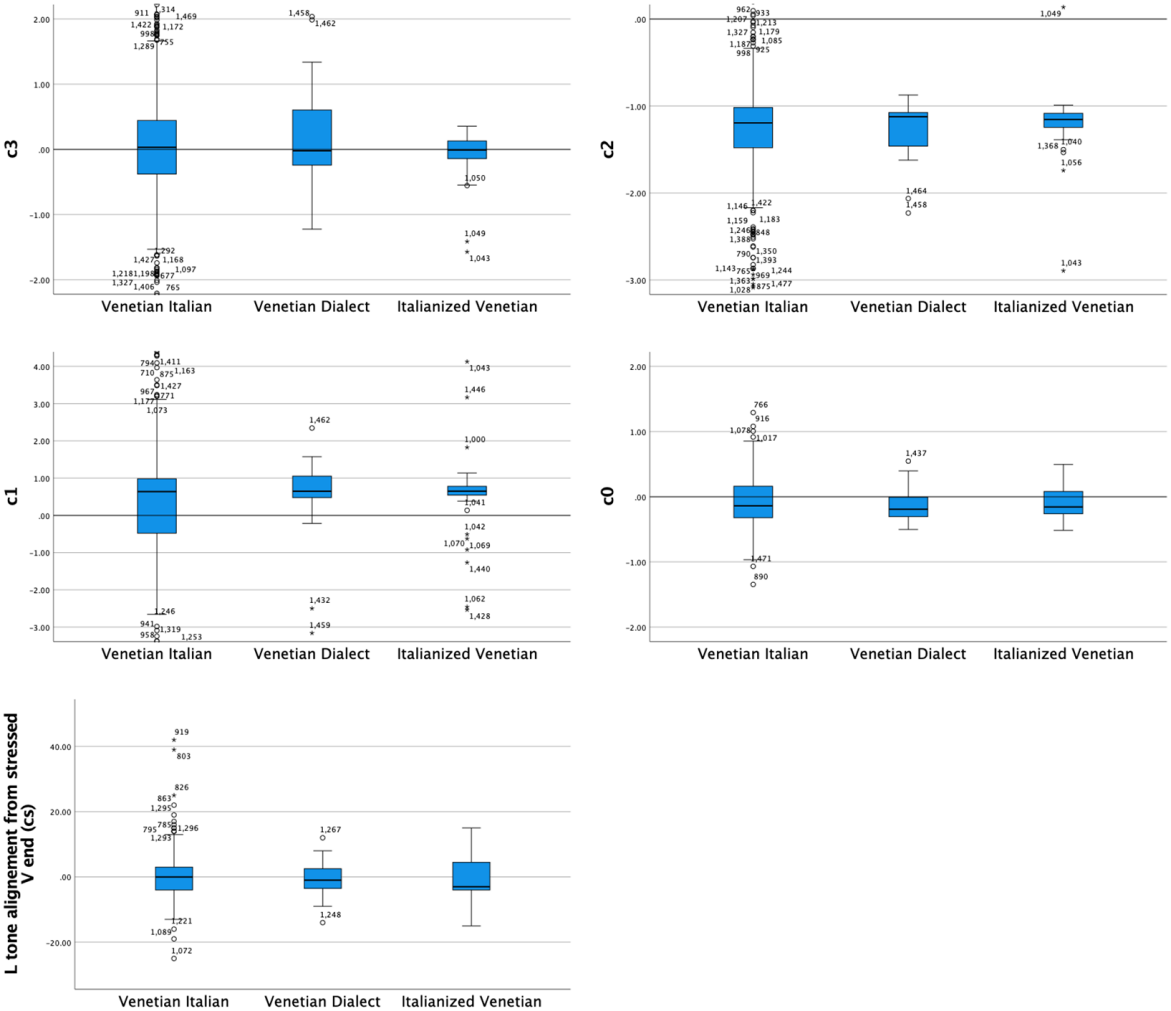
Comparison of the four coefficients (top two rows) and the L tone alignment in declaratives between Venetian Italian, Venetian Dialect, and Italianized Venetian.

A Kruskal–Wallis H test compared the alignment of the L tone from the end of the stressed vowel (Figure 9 bottom row) and the four coefficients (i.e., c3 to c0; Figure 9 top two rows) in Venetian Dialect (*N* = 23), Italianized Venetian (*N* = 25), and Venetian Italian (*N* = 785). This test showed that there was no statistically significant difference in L alignment nor in any of the four coefficients, that is, between any pair of varieties; alignment: χ^2^(2) = 0.302, *p* = .860, with a mean rank alignment score of 263.37 for Venetian Italian, 251.57 for Venetian Dialect and 253.13 for Italianized Venetian; c3: χ^2^(2) = 0.690, *p* = .708, with a mean rank c3 score of 418.13 for Venetian Italian, 428.89 for the Venetian Dialect and 387.78 for Italianized Venetian; c2: χ^2^(2) = 0.961, *p* = .618, with a mean rank c2 score of 415.00 for Venetian Italian, 419.27 for Venetian Dialect and 452.77 for Italianized Venetian; c1: χ^2^(2) = 0.494, *p* = .781, with a mean rank c1 score of 415.40 for Venetian Italian, 445.00 for Venetian Dialect and 427.72 for Italianized Venetian; c0: χ^2^(2) = 0.311, *p* = .856, with a mean rank c0 score of 417.71 for Venetian Italian, 392.29 for Venetian Dialect and 420.56 for Italianized Venetian.

The results indicate no significant difference in any of the intonation variables tested. Therefore, in the subsequent comparison presented in Section 4, all three Venetian dialects will be treated as one group which will be compared with Cretan and Athenian.

## 4 Results

Overall, comparisons of the declarative and polar question tunes among the three language varieties revealed similarities between Cretan and Venetian in shape and alignment characteristics. Quantitative results on the declarative tune comparisons are presented below in 4.1 and on the polar question tune comparisons in Section 4.2.

### 4.1 The declarative tune

The analysis of the declarative tunes indicated an absence of striking difference in the tune shape. This is not unexpected as declarative utterances in all three varieties can be broadly characterized as falls. However, the finer details of those falling contours are variety-dependent, with both the value of the cubic and the quadratic coefficients contributing to more subtle variations in the fall shape. A detailed analysis is presented in Section 4.1.1. In contrast, the alignment analysis presented in Section 4.1.2 produced a stronger result, showing Cretan to be much more similar to Venetian than to Athenian.

#### 4.1.1 Shape coefficients

**Figure 10.**
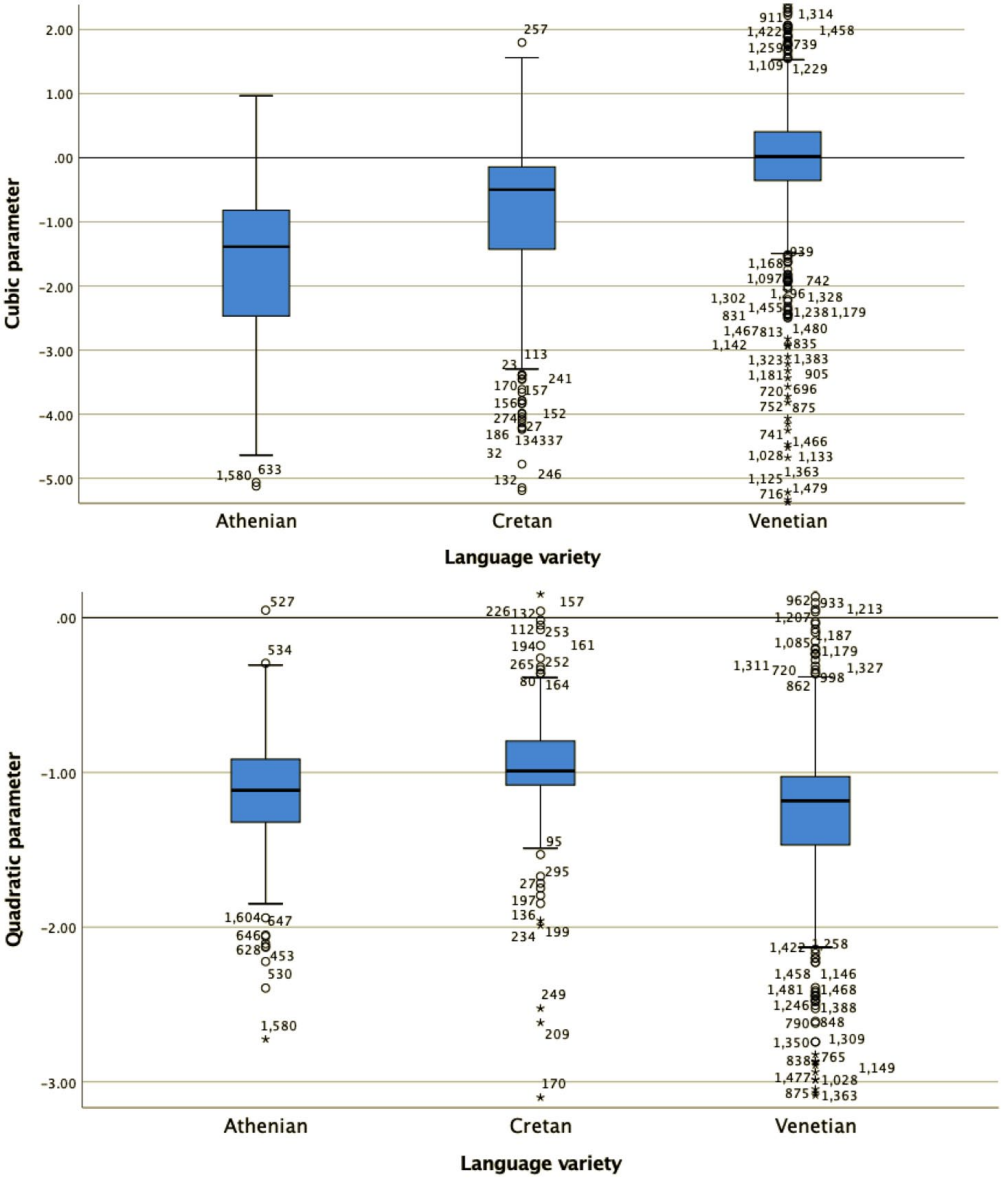
The distribution of values of the cubic c_3_ (top) and quadratic coefficient c_2_ (bottom) in the three language varieties in declaratives.

**Figure 11.**
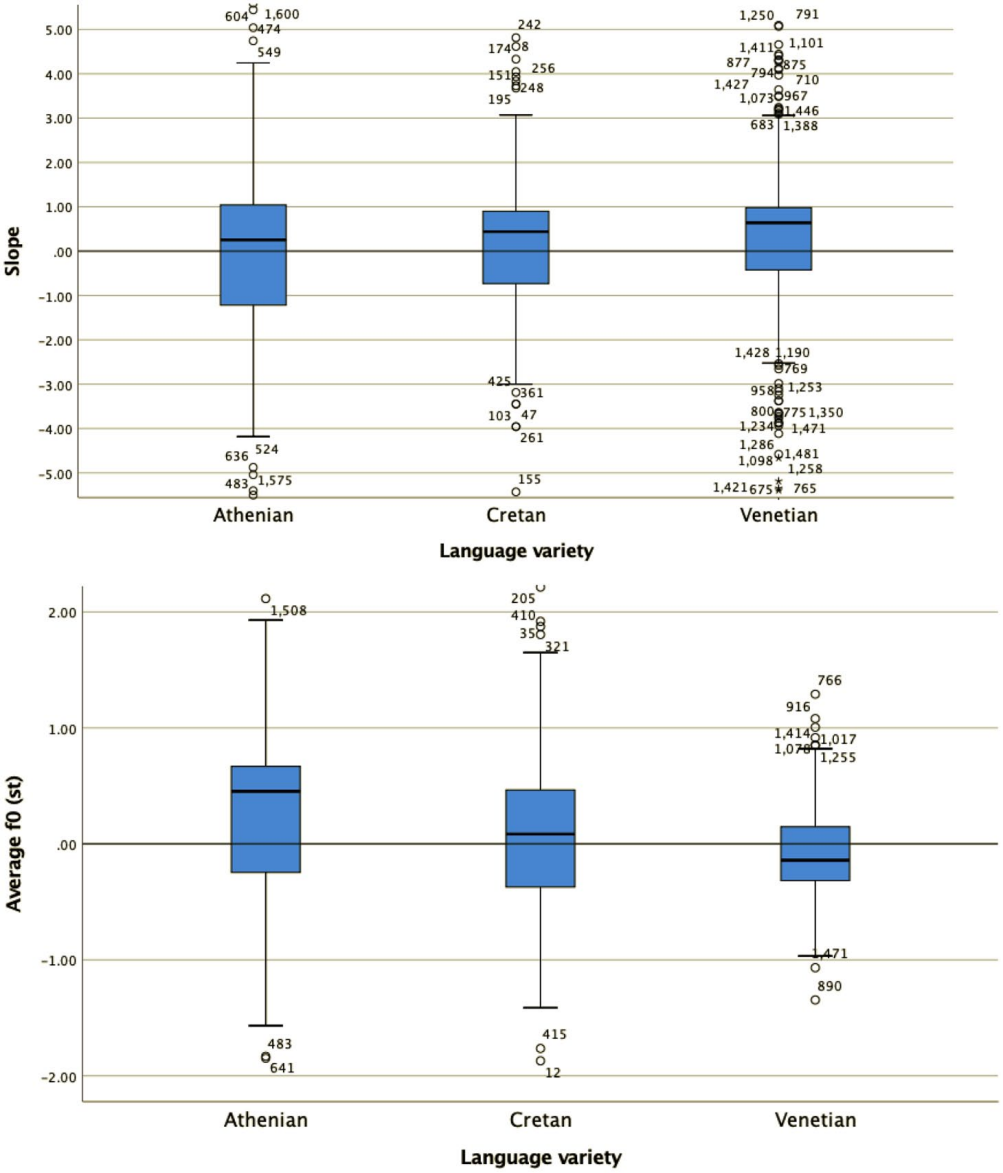
The distribution of values of the slope c_1_ (top) and average F0 coefficient c_0_ (bottom) in the three varieties in declaratives.

For all four shape coefficients (c_0_, c_1_, c_2_, and c_3_, the coefficients for the constant (or average F0), slope, quadratic and cubic, term, respectively) as well as for the alignment of the L trough with the end of the stressed vowel, a Kruskal–Wallis test provided very strong evidence of a difference between the mean ranks of at least one pair of groups (Figures 10 and 11): c_0_ χ^2^(2) = 483.9, *p* < .001; c_1_ χ^2^(2) = 192.29, *p* < .001; c_2_ χ^2^(2) = 22.94, *p* < .001; c_3_ χ^2^(2) = 93.35, *p* < .001. Dunn’s pairwise tests were carried out for the three pairs of language varieties. There was very strong evidence (*p* < .001, adjusted using the Bonferroni correction) of a difference between all three pairs of language varieties for coefficients c_0_, c_1_, and c_2_. For alignment, there was also very strong evidence of a difference (*p* < .001 between Athenian and Cretan and between Athenian and Venetian; *p* < .005 between Cretan and Venetian). For c_3_, there was very strong evidence (*p* < .001, adjusted using the Bonferroni correction) of a difference between Athenian and Venetian and between Cretan and Venetian. There was no evidence of a difference between Athenian and Cretan.

The mean and median coefficient values of each language variety are also important for the interpretation of the results. For example, the cubic coefficient (Figure 10, top panel) models the pitch contour shape as a wave. The Venetian curves have cubic coefficients (c_3_) that cluster around zero (median c_1_ = 0.02), modeling the shape as an F0 plateau, while the Athenian curves, with mostly negative cubic coefficient values (median c_3_ = –1.39), are modeled as fall-rise-fall movements. Cretan is somewhere in between Athenian and Venetian. Its cubic coefficient values (median c_3_ = –0.5) have smaller negative values than Athenian, indicating that the fall-rise-fall movements are smaller. The values of the other three coefficients do not show big differences (for Athenian, Cretan, and Venetian respectively, quadratic: –1.12, –0.99, –1.18; slope: 0.25, 0.44, 0.64; constant: 0.45, 0.09, –0.14), so they will not be discussed further here.

#### 4.1.2 Alignment

**Figure 12.**
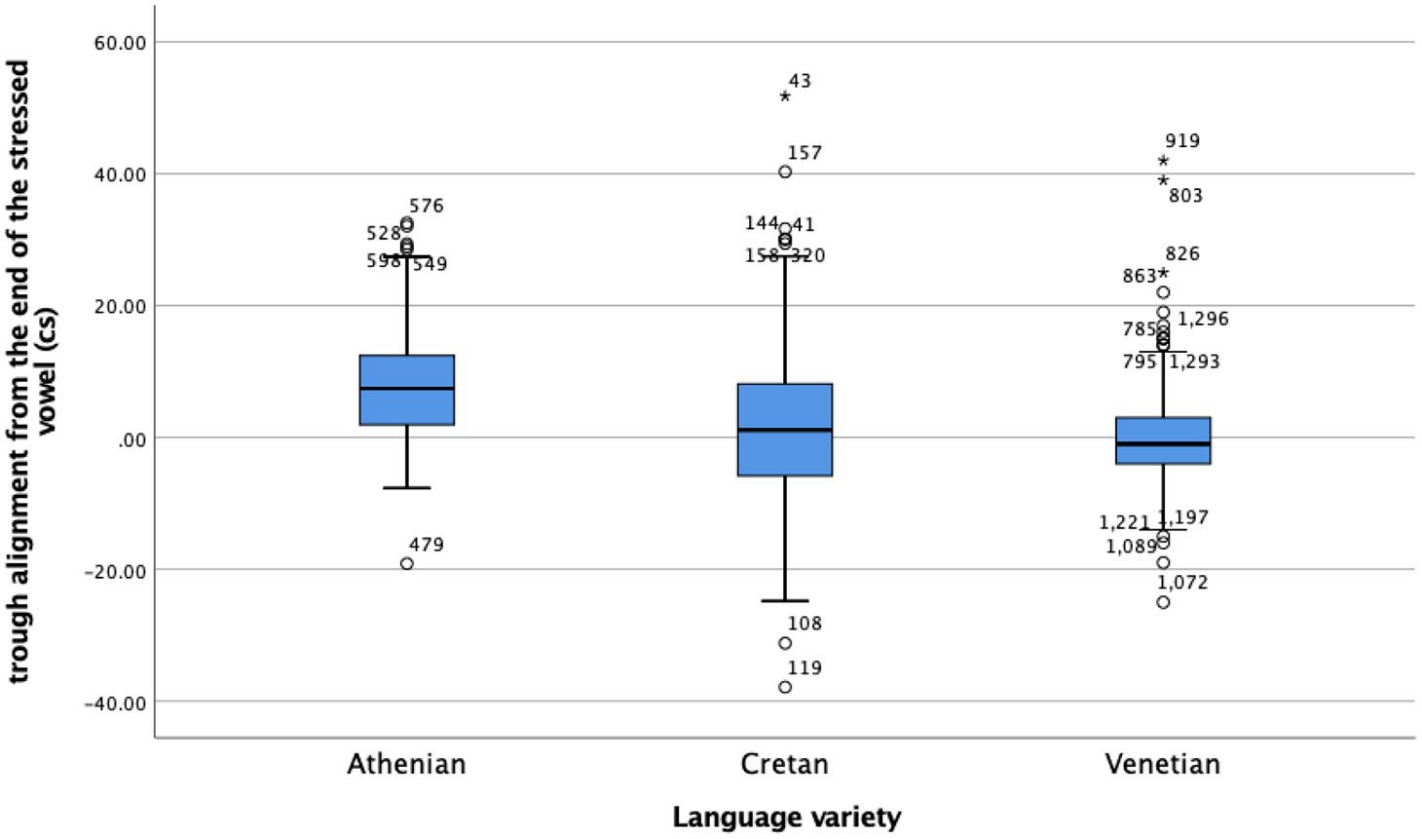
Alignment of the L trough from the end of the nuclear vowel. Positive values indicate that the trough is later than the end of the vowel.

Regarding alignment (Figure 12), the median distance of the L was 74 milliseconds after the end of the nuclear vowel in Athenian, while the L in Cretan and Venetian was closely coupled with the right edge of the nuclear vowel, 11 milliseconds after it in Cretan and 10 milliseconds before it in Venetian, a very small difference between Cretan and Venetian.

**Figure 13.**
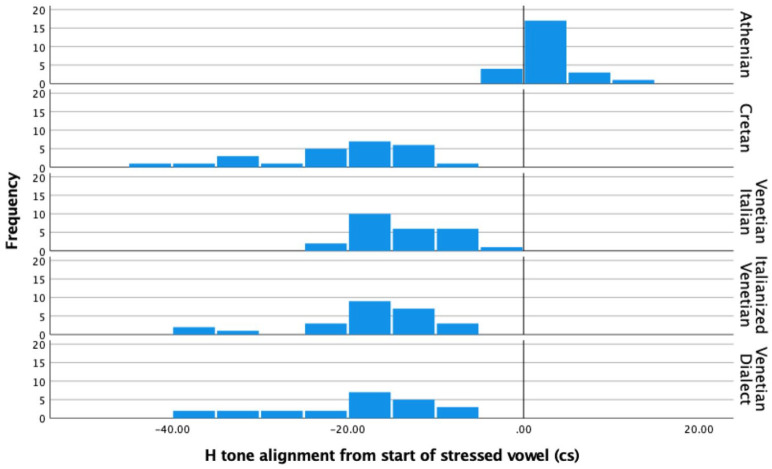
H tone alignment from the start of the stressed vowel in declaratives. Shown from top to bottom: Athenian, Cretan, Venetian Italian, Italianized Venetian, and Venetian Dialect. Time *t* = 0 in the x-axis represents the start of the stressed vowel.

**Figure 14.**
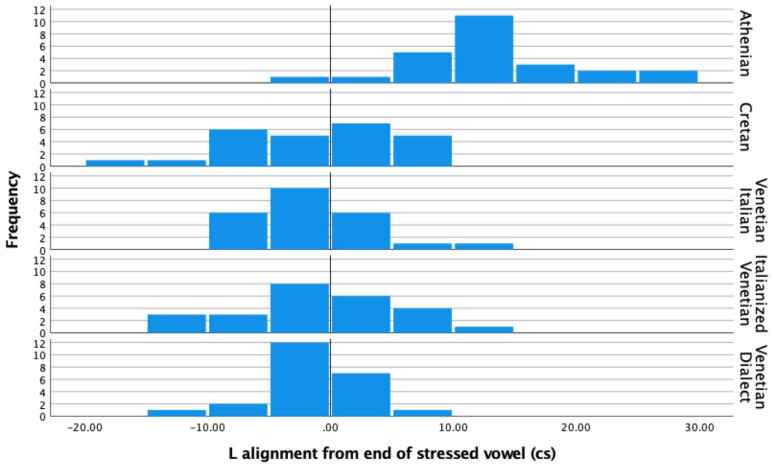
L tone alignment from the end of the stressed vowel in declaratives. Shown from top to bottom: Athenian, Cretan, Venetian Italian, Italianized Venetian, and Venetian Dialect. Time *t* = 0 on the x-axis is the end of the stressed vowel.

We further submitted a balanced, randomly selected subset of the declarative data to a more detailed alignment analysis (Athenian *N* = 25; Cretan *N* = 25; Venetian Italian *N* = 25; Italianized Venetian *N* = 25; Venetian Dialect *N* = 23). This analysis involves both the H peak (Figure 13) and L trough (Figure 14). As discussed in 1.3, in Venetian and Cretan there is an H tone in the prestress vowel before the trough which occurs in the stressed/nuclear vowel. In contrast, in Athenian the H tone occurs within the stressed vowel and the L tone on average occurs well after the end of the stressed vowel. Data from the three Venetian varieties are shown (and statistically compared) separately to further corroborate the claim that they pattern similar to one another.

An ANOVA test showed there was a significant effect of language variety on the alignment of the H tone (Figure 13) for the five language varieties, *F*(4, 118) = 45.58, *p* < .001. A post Hoc Tukey HSD test indicated that the mean H tone alignment with respect to the beginning of the nuclear vowel in Athenian (*M* = 2.78 cs, *SD* = 0.6) was significantly different from the alignment in all other language varieties. All other comparisons revealed no significant differences (Cretan *M* = –20.79 cs, *SD* = 9.2; Venetian Italian *M* = –14.31 cs, *SD* = 5.15; Italianized Venetian *M* = –17.31 cs, *SD* = 7.33; Venetian Dialect *M* = –19.74, *SD* = 9.06).

An ANOVA test showed a significant effect of language variety on the alignment of the L tone (Figure 14) for the five language varieties, *F*(4, 118) = 4.31, *p* = .003. A post Hoc Tukey HSD test indicated that the mean L tone alignment with respect to the end of the nuclear vowel in Athenian (*M* = 13.22 cs, *SD* = 6.9) was significantly different from the alignment in all other language varieties. All other comparisons revealed no significant differences (Cretan *M* = –1.4 cs, *SD* = 7.5; Venetian Italian *M* = 4.53 cs, *SD* = 31.14; Italianized Venetian *M* = –0.58 cs, *SD* = 6.9; Venetian Dialect *M* = –1.75 cs, *SD* = 4.2).

#### 4.1.3 Summary of declarative F0 contours

**Figure 15.**
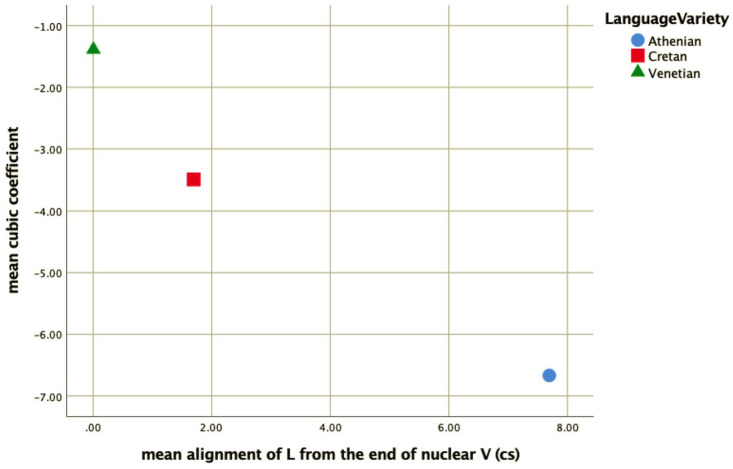
Mean values of the cubic coefficient plotted against the mean alignment of the L from the end of the nuclear vowel for Athenian (circle), Cretan (square), and Venetian declaratives (triangle).

The similarity of the Cretan to the Venetian declarative pitch contour shape is indicated by the distribution of the cubic coefficient c_3_ and the L alignment. In other words, the shape of the Athenian declarative pitch contours as modeled by the cubic coefficient is a fall-rise-fall contour, as illustrated in the observed contour in Figure 1, while the Cretan and the Venetian declarative contour is a fall from an H peak with a low F0 plateau, as illustrated in the observed shape in Figures 2 and 3. In addition, the end of the fall (the L) is typically aligned with the nuclear vowel in Cretan and Venetian but after the end of this vowel in Athenian. Figure 15, a plot of the mean values of the cubic coefficient and alignment parameter, depicts this similarity, with the Cretan values closer to Venetian than to the Athenian ones.

### 4.2 The polar question tune

The analysis of the polar question tunes indicated an even stronger similarity between Cretan and Venetian than was found in the analysis of declaratives. Our data revealed no significant difference between Cretan and Venetian in the alignment of the H peak, as well as for three of the four shape coefficients, whereas the H peak alignment and the same three shape coefficients were significantly different from Athenian.

**Figure 16.**
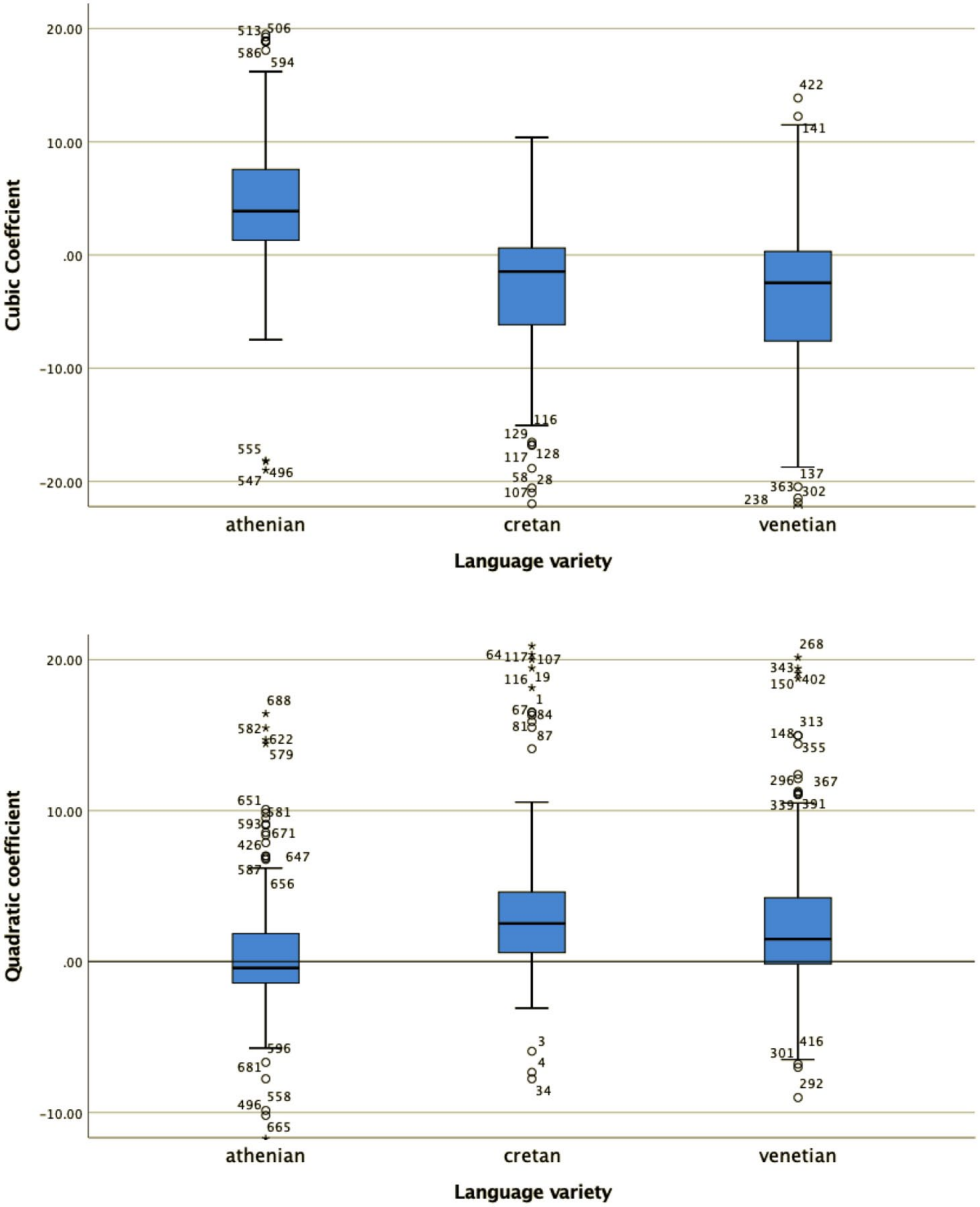
The distribution of values of the cubic c_3_ (top) and quadratic coefficient c_2_ (bottom) in the three language varieties in polar questions.

**Figure 17.**
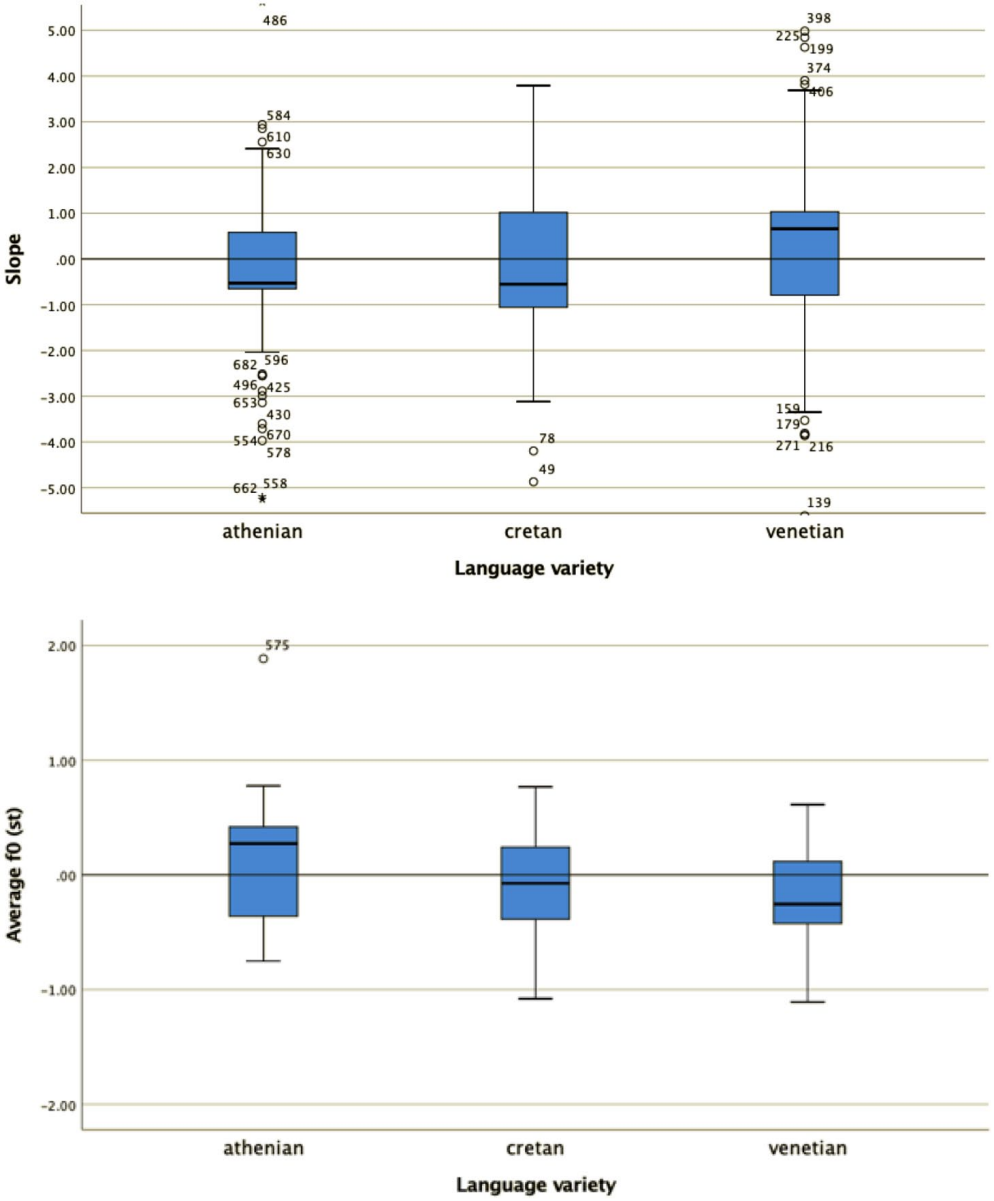
The distribution of values of the slope c_1_ (top) and average F0 coefficient c_0_ (bottom) in the three language varieties in polar questions.

**Figure 18.**
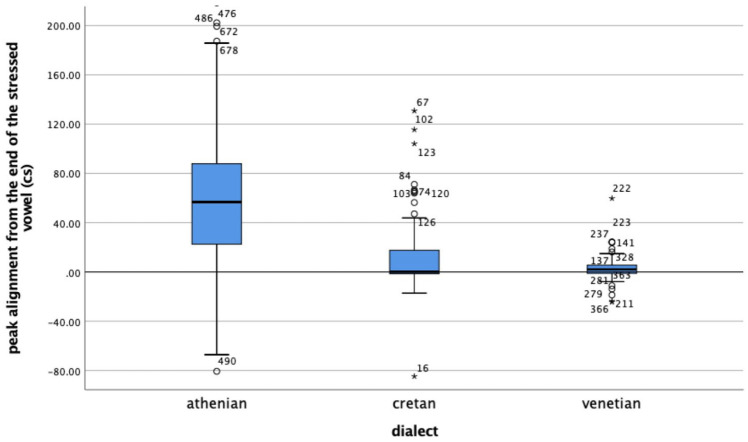
Alignment of the H peak from the end of the nuclear vowel. Positive values indicate that the peak is later than the end of the vowel.

Specifically, for all four shape coefficients,c_0_, c_1_, c_2_, c_3_ (Figures 16 and 17) as well as the alignment of the H from the end of the nuclear vowel (Figure 18), a Kruskal–Wallis test provided a very strong evidence of a difference between the mean ranks of at least one pair of groups. Dunn’s pairwise tests were carried out for the three pairs of language varieties, c_0_ χ^2^(2) = 251.66, *p* < .001; c_1_ χ^2^(2) = 65.72, *p* < .001; c_2_ χ^2^(2) = 16.79, *p* < .001; c_3_ χ^2^(2) = 50.79, *p* < .001; alignment χ^2^(2) = 218.63; *p* < .001. There was very strong evidence (*p* < .001, adjusted using the Bonferroni correction) of a difference between Athenian and Cretan (*p* < .001) and between Athenian and Venetian (*p* < .001) for three of the four shape coefficients (c_0_, c_1_, and c_2_), as well as for alignment. There was no evidence of a difference between Cretan and Venetian for three of the four coefficients (c_0_, c_1_, and c_2_) or for alignment. For coefficient c_3_, there was very strong evidence of a difference between Athenian and Venetian (*p* < .001, adjusted using the Bonferroni correction), and between Cretan and Venetian (*p* < .019), but no evidence of a difference was found between Athenian and Cretan.

**Figure 19.**
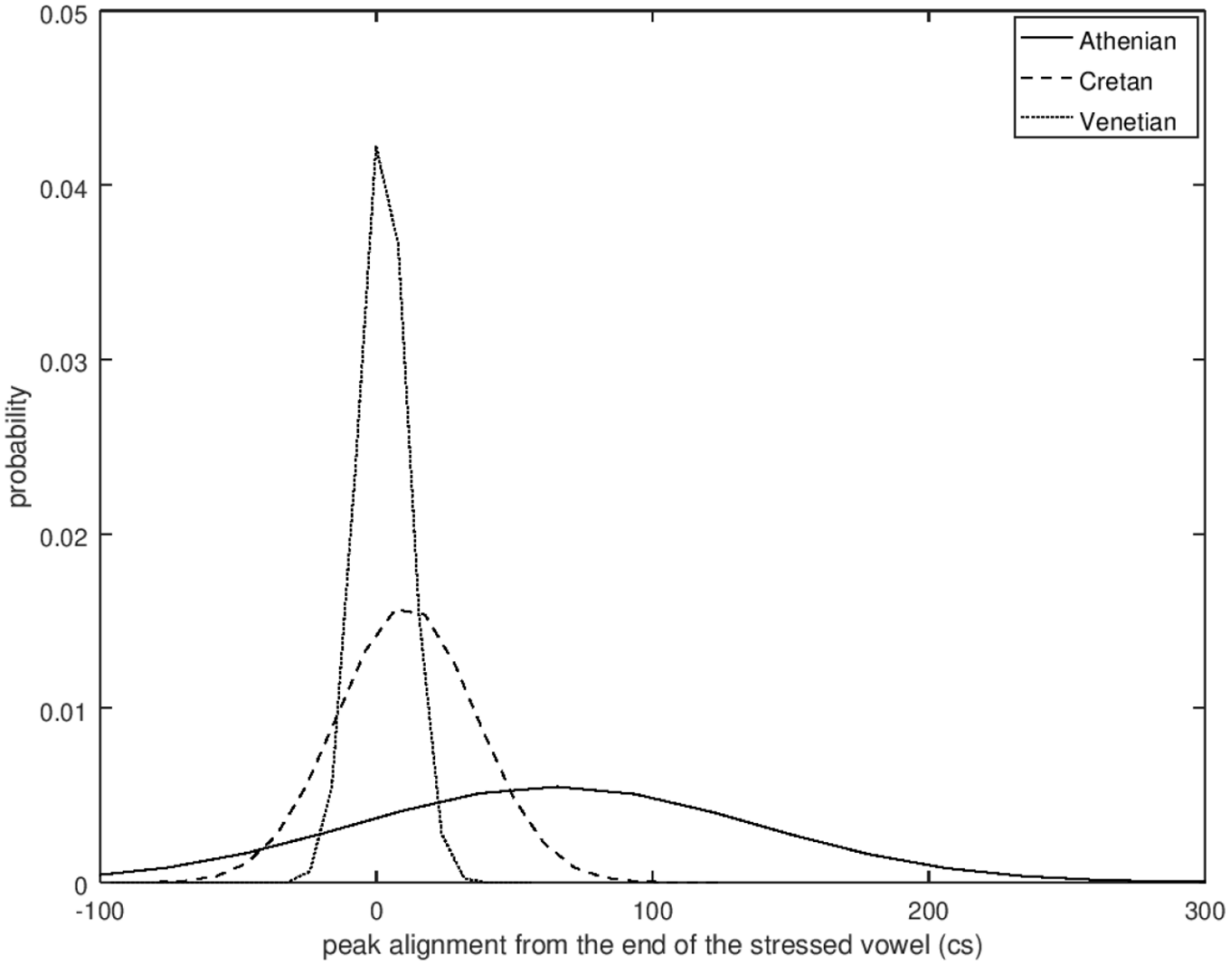
Probability density estimates of the distribution of the peak alignment values in the three language varieties.

The mean and median coefficient values indicate that the polar question pitch contour of Cretan resembles that of Venetian and is different from the Athenian. The cubic coefficient of Cretan and Venetian has a negative median value (–1.46 for Cretan and –2.45 for Venetian), while in Athenian it is positive (3.72), indicating that the wave shape is similar for Cretan and Venetian but flipped about the horizontal axis for Athenian. The quadratic coefficient is negative in Athenian (median –0.45), indicating it opens downward but positive in the other two (Cretan median: 2.51, Venetian median: 1.48), indicating it opens upward. The peak alignment also provides very strong evidence of similarity between Cretan and Venetian. The median distance of the H after the end of the nuclear vowel was 1.8 milliseconds in Cretan and 2.1 milliseconds in Venetian. In contrast, the 567.9 milliseconds median distance from the end of the nuclear vowel in Athenian is a large difference from Cretan (see also Figure 19 which depicts the different distributions of the H alignment in the three language varieties).

**Figure 20.**
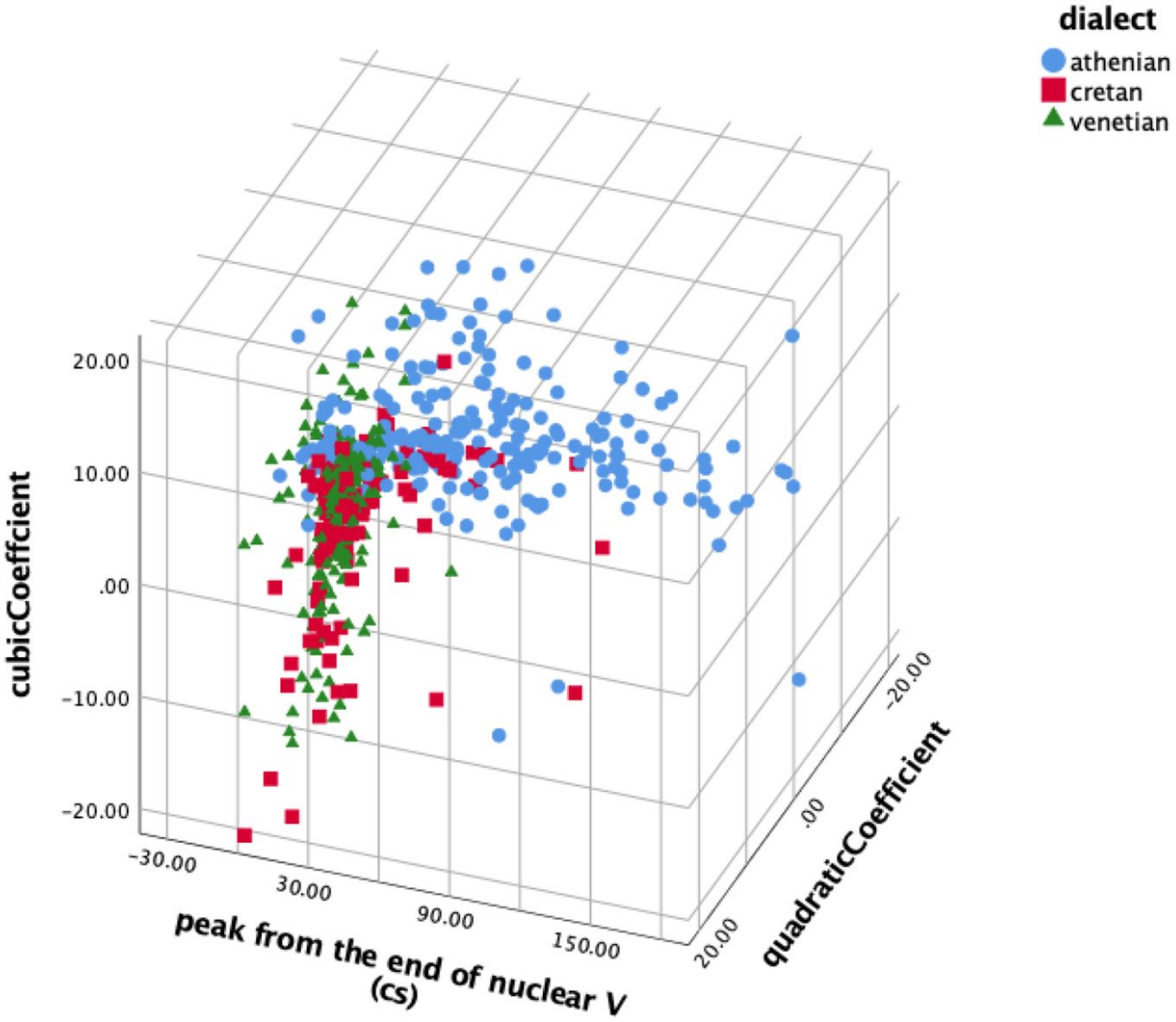
The cubic coefficient plotted against the quadratic coefficient and mean alignment of the H peak from the end of the nuclear vowel for Athenian (circle), Cretan (square), and Venetian declaratives (triangle).

Overall, the similarity of the Cretan to the Venetian polar question pitch contour shape is indicated by four of the five parameters that we examined. Figure 20 which plots the distribution of three of these parameters shows that the Cretan and Venetian data points largely overlap in the space defined by the cubic, quadratic, and alignment parameters, while the Athenian data points are mostly separated from Cretan and Venetian.

## 5 Discussion

The results presented earlier confirm our predictions that the intonation patterns of Cretan Greek declarative and polar question tunes are similar to those of Venetian, highlighting the robustness of contact effects almost three and a half centuries after regular contact ceased. Similar findings have been reported in the literature about the persistent effects of language contact on the diachronic development of intonation (Baltazani et al., 2019a, 2019b; Bullock, 2009; Colantoni & Gurlekian, 2004) but not for contact that ceased as long ago as in this case.

Similarities between Cretan and Venetian and differences between Cretan and Athenian in the shape of the F0 contours were found for all four shape coefficients (c_0_ to c_3_) in polar questions, but not so in declaratives, something that is expected given that declarative utterances in all the three varieties can be broadly characterized as falls. Nevertheless, even in declarative utterances, examination of the fine details revealed subtle variations in the fall shape between the varieties, suggesting that although F0 contours are different in the three varieties, the quantitative description of Cretan contours places them closer to Venetian than Athenian ones. Clear similarities between Cretan and Venetian in the shape of the F0 contours were found in polar questions, where no significant differences were found for three of the four coefficients we examined.

Even more compelling evidence of similarities between Cretan and Venetian was provided by the alignment analysis. No significant difference was found between these two varieties in the pattern of alignment of the peaks and troughs in declaratives and the peaks in polar questions. There were only a few milliseconds of difference in the mean duration of the interval from the nuclear L tone in declaratives and the nuclear H tone in polars to the end of the nuclear vowel. In contrast, the corresponding L and H tonal minima and maxima in Athenian tunes were aligned further away from the end of the nuclear vowel, especially in polar questions, where the H peak appeared 570 milliseconds later. An additional, more detailed analysis of declaratives revealed that the H in Athenian was aligned with the nuclear vowel, in line with previous reports of a nuclear H* or H*+ L in Athenian (Arvaniti & Baltazani, 2005; Baltazani, 2002, 2003; Lohfink et al., 2019). In contrast, there was an H tone that was consistently aligned within the pre-accentual vowel in Cretan and Venetian, suggesting a leading tone to a bitonal H + L* nuclear accent. This finding is in agreement with a previous report of a H + L* nuclear accent in Cretan (Baltazani & Kainada, 2019) but differs somewhat from the proposal in Dévis-Herraiz (2007) about Venetian, who describes the declarative nuclear tone in this variety as L*. This difference could be attributed to the fact that the speakers in the Dévis-Herraiz (2007) study were bilinguals (L1 Italian and L2 Spanish), which means that the results in the two studies are not directly comparable.

These findings are also relevant to regional intonational variation. They bridge a gap in our knowledge about the intonation of northern Italian varieties, as very little work exists on the intonation of Venetian, Venetian Italian, and Greek regional varieties. There has been very little research into the intonation of Cretan, with only one previous study of the declarative tune (Baltazani & Kainada, 2019), to our knowledge. As for Venetian, language contact between the variety of Italian spoken in Venice and the Venetian dialect has resulted in a continuum between the two, with frequent code mixing (Grassi, 1993). Previous studies have described the lexical, morphological, segmental, and intonational features that characterize the Venetian dialect (e.g., Canepari, 1976, 1986), but work comparing the dialect and the variety of Italian spoken in Venice is scarce and has focused on morphological and segmental features (Ferguson, 2007). An analysis of the utterances in our corpus based on these markers of linguistic varieties indicated no difference between them in intonation. This finding accentuates the fact that a difference in certain aspects of grammar does not necessarily mean a difference in all aspects of grammar.

From a methodological point of view, this is a corpus-based study of natural speech data, rather than laboratory speech of controlled materials in which utterances are designed to differ minimally only in the parameters of interest. The utterances we examined have different lengths, different syntactic makeup and complexity, and are a collection of different types of speech, for example, spontaneous conversations, interviews, television and radio programs, map tasks, and spontaneous narratives. Functional data analysis of F0 contours (here using polynomial basis functions) allows us to deal with the variability in the materials we analyzed and automate most steps in the investigation of these tunes. Avoiding manual analysis as far as possible makes the process faster and more accurate. Part of the automatic analysis was the location of F0 peaks and troughs analytically through modeling with the added benefit of the freedom to handle utterances regardless of their segmental makeup. The inability of pitch tracking algorithms to estimate F0 during voiceless stretches has meant that most prior work on intonation relies on the use of rather unnatural laboratory utterances composed to contain mostly voiced segments.

Our finding that Cretan Greek intonation closely resembles Venetian intonation and is markedly different from Athenian Greek supports our hypothesis that this similarity is the result of contact between Venetian and Cretan Greek. Of course, we cannot be *sure* that contact is the cause of the striking similarities–they might simply be due to a cross-linguistically common prosodic “type.” It is possible that Cretan Greek happened to be similar to Venetian a priori, although that begs the question as to why Cretan intonation is different from that of other Greek varieties. The statistical results show that the probability of accidental or coincidental similarity is extremely low. The contact hypothesis, on the other hand, does predict the similarities and significant differences which we in fact found.

The surviving effects of Venetian influence on the intonation of Cretan Greek, although the Venetian-Greek cohabitation in Crete ended three and a half centuries ago, gives rise to the question: how can such contact-induced changes last for so long after the period of contact? Cretan Greek appears to have been receptive enough to be influenced by Venetian and yet at the same time, somewhat paradoxically, conservative enough to resist change after having borrowed these two tunes.

The following related ideas suggest themselves. The first is that although in general change and development are in a broad sense always ongoing, periods of change are punctuated with periods of stability, as change and development usually come about in specific historical and social circumstances (Nicholls, 2003, p. 287). Although Nicholls mainly considers nonphonetic linguistic changes such as lexical and grammatical changes, there is growing evidence from phylogenetic modeling of sound change that it must be punctuated, not gradual; Hruschka et al. (2015), for example, estimates the rate of historical sound change at approximately one regular sound change per 385 years, a time period so long that most generations *cannot* experience any lasting phonological changes. Supporting evidence about the effect of historical and social circumstances on intonation change is presented by the Greek varieties spoken in the Asia Minor peninsula. These varieties were influenced by Turkish, due to nine centuries of Greek-Turkish co-habitation, but Baltazani et al. (2020), a diachronic study, revealed that the Turkish influence on the intonation of Greek diminished just one generation after the 1923 Greek-Turkish population exchange due to the change in their sociolinguistic circumstances. In Crete, there was a long period of contact with Venice, first for trade and later for a long period of Venetian dominion. During this time, the Greek spoken in Crete took on Venetian lexical features (Horrocks, 2010) and also, we have shown, intonational ones.

Second, the local policy of the Ottomans in Crete, who did not colonize it during their occupation of the island, meant little culture mixing during this period. This is likely to have preserved the language patterns established in Crete during the Venetian period. Third, even during and after the Ottoman period, Greek-speaking Cretans continued to have close cultural and trading contacts with Venice and the Venetian Adriatic. Large-scale contact due to cohabitation between populations ceased, but small ways in which the ties between Venetians and Cretans were maintained must have been sufficient to preserve its linguistic outcomes. The speech patterns of which Cretans are proud, and which contribute to their distinct identity in Greece, appear to have their origins in part in centuries of contact with their Mediterranean neighbors. As there are still very few studies of how historical contact affects intonational similarities and differences across language varieties, this being one of the first attempts to quantify such similarities, and further research is undoubtedly needed.

## Appendix A

### Sources of audio recordings

#### Athenian Greek

1. Academy of Athens, http://www.academyofathens.gr/en. Digitized archival tape recordings shared with us (not on the public internet).
2. Vocalect, http://www.vocalect.eu/?lang=en.
3. Radio show “Ellinofrenia,” 7 December 2015, https://ellinofreneianet.gr/radio.
4. 50 Languages.com, https://www.50languages.com/phrasebook/en/el/.
5. Speak Greek, Aristotle University of Thessaloniki http://speakgreek.web.auth.gr/wp/.

#### Venetian Italian

1. CLIPS: Corpora e Lessici dell’Italiano Parlato e Scritto. http://www.clips.unina.it/it/
2. Vivaldi: Vivaio Acustico delle Lingue e dei Dialetti d’Italia, https://www2.huberlin.de/vivaldi/?id=0001&lang=it
3. Rai Radio 3: Pantagruel, Venezia, 4 April 2019. https://www.raiplayradio.it/audio/2019/07/RaiTv-Media-Audio-Item-7bb7e039-1dc3-42e9-b5cc-7d32de3055d7.html
4. Archivio del parlato—La tramontana e il sole, https://www.lfsag.unito.it/ark/table_ita.html

#### Cretan Greek

1. Folklore museum archive, University of Athens, http://www.phil.uoa.gr/biblio8ikh-ergastiria-moyseia/laografiko-moyseio-arxeio.html Digitized archival tape recordings shared with us (not on the public internet).
2. Academy of Athens http://www.academyofathens.gr/en. Digitized archival tape recordings shared with us (not on the public internet).
3. The *Greek Dialects Archive*, School of English, Aristotle University of Thessaloniki, http://www.enl.auth.gr/greekdialects/preface.htm
4. Ανέκδοτα - Στάθης Μονιάκης - Γιώργος Βιτώρος. YouTube https://www.youtube.com/watch?v=IOFBbfzJgk0
5. Vocalect http://www.vocalect.eu/?lang=en

## Appendix B

### Criteria for the classification of the Venetian varieties

Based on a number of segmental, morphological, and lexical criteria for dialect features (see also Canepari, 1976; Ferguson, 2007), one of the authors, a native speaker of northern Italian (Lake Garda), familiar with the speech of the Veneto region, classified the utterances as belonging into one of the categories: (1) Venetian Italian (*N* = 764), (2) Italianized Venetian (*N* = 41), and (3) Venetian Dialect (*N* = 28); see Cecchinato (2017) for a similar approach. Our data contains no utterances in Standard Italian. All our informants come from the Venice area. Utterances in Category 3 contain only dialectal syntactic, lexical, and segmental traits. Utterances in Category 2 contain some dialectal syntactic, lexical, and segmental traits and some nondialectal (i.e., Standard Italian) ones. Utterances in Category 1 contain no syntactic, lexical, or segmental dialectal traits.

**Table:**

Criteria	Venetian Dialect traits	Non-dialectal traits
**Phonetic**		
Gemination	No, e.g., *soto* [soto] “under”	Yes, *sotto* [sot: o] “under”
Evanescent /l/	[a pipa] “the pipe”	[la pipa] “the pipe”
Rhotic consonants	Retroflex flap, [baɽə] “bar”	Alveolar tap, [baɾə] “bar”
**Morphological**		
1^st^ person pronoun	*mi* obligatory	*io* optional
/a/ apheresis	(*in fianco*) *‘l* “next to”	(*in fianco*) *al* “next to”
**Lexical**		
Verb “to have” infinitive	*gáver*	*avere*
Verb “to have” 1st person	*go*	*ho*
“that”	*queo*	*quello*
“drawing”	*dexégno*	*disegno*

## Notes

1. *Intonation and diachrony: how language contact shapes intonational patterns*, supported Grant ES/R006148/1 from the UK Economic and Social Research Council.

2. Although MP3 and MP4 files typically use audio compression, with some loss of fidelity to the original recorded signal, Fuchs and Maxwell (2016) found that mp3 “compression rates between 56 and 320 kbps show relatively small mean errors of 2% or less, with median errors well below 0.5% . . . . mp3 compressed data is viable for the analysis of F0” (p. 525). They found that measurement errors in mp3-compressed data were greatest of all in consonants, for example, the perturbations to F0 caused by obstruents, which are not relevant to the present study. Indeed, the variation in measured F0 due to different estimation algorithms and analysis parameters can be far greater than the 0.5% to 2% error rate arising from mp3 compression.

3. An analysis of variance test with each of the first four coefficients, as well as the alignment values as dependent variables and dialect, speaker age group (young, mid, and old), speaking style (conversational, narrative, and semi-spontaneous), and gender (M, F) as factors showed that only dialect was a significant factor.

4. This analysis was not possible for polar questions, due to the lack of tokens in Venetian Dialect.
